# Person-centred care and the work-related health and job satisfaction of health and social care professionals: protocol for a prospective longitudinal cohort study combined with qualitative studies (the PCC@Work project)

**DOI:** 10.1186/s12913-024-11148-z

**Published:** 2024-05-30

**Authors:** Cornelia van Diepen, Qarin Lood, Kristoffer Gustavsson, Malin Axelsson, Monica Bertilsson, Gunnel Hensing, Andreas Fors

**Affiliations:** 1 Erasmus School of Health Policy & Management, Erasmus University Rotterdam, Rotterdam, Netherlands; 2 Centre for Person-Centred Care (GPCC), University of Gothenburg, Gothenburg, Sweden; 3 Institute of Neuroscience and Physiology, Department of Health and Rehabilitation, Sahlgrenska Academy, University of Gothenburg, Gothenburg, Sweden; 4 Centre for Ageing and Health - AgeCap, University of Gothenburg, Gothenburg, Sweden; 5 Institute of Health and Care Sciences, Sahlgrenska Academy, University of Gothenburg, Goteborg, Sweden; 6 Department of Care Science, Faculty of Health and Society, Malmö University, Malmö, Sweden; 7 School of Public Health and Community Medicine, Institute of Medicine, Sahlgrenska Academy, University of Gothenburg, Gothenburg, Sweden; 8 Research, Education, Development and Innovation, Region Västra Götaland, Primary Health Care, Gothenburg, Sweden

**Keywords:** Study protocol, Person-centred care, Health and social care professionals, Cohort study, Job satisfaction, Stress of conscience, Psychosocial work environment, Well-being

## Abstract

**Background:**

The interplay of ethical stress, heavy workloads, and job dissatisfaction poses challenges to both the recruitment and retention of health and social care professionals. Person-centred care, rooted in ethical principles, involves collaborative care, and is expected to improve care and job satisfaction. However, prior research on the impact of person-centred care practices on professionals’ work-related health and job satisfaction has yielded mixed results, and most studies emanate from residential care. Understanding how person-centred care practices influence health and social care professionals across different care settings thus requires further exploration through rigorous methodology. The overall aim of PCC@Work is to follow, describe, assess, and explore the impact of person-centred care practices in hospital wards, primary care centres and municipal care on health and social care professionals’ work-related health and job satisfaction.

**Methods:**

PCC@Work is designed as a prospective, longitudinal cohort study combined with qualitative studies. A web-based questionnaire will be distributed on five occasions within two years to health and social care professionals in the three care settings. In addition, focus groups and interviews will be conducted with a selection of health and social care professionals to explore their experiences of work-related health and job satisfaction in relation to person-centred practices.

**Discussion:**

PCC@Work will highlight some of the knowledge gaps on the impact of person-centred care practices regarding work-related health and job satisfaction of health and social care professionals. The uniqueness of the project lies in the multi-method design, combining a prospective longitudinal cohort study with qualitative studies, and the involvement of various professions and settings. This means we will be able to provide a comprehensive and representative understanding of person-centred care practices as a critical component for effective change in the working conditions of health and social care.

## Background

There is a growing interest in person-centred care (PCC) since authorities, such as the World Health Organization (WHO), have called for enabling patients to engage in their care and treatment [1]. PCC has also been endorsed by health and social care professionals and patient organisations [2, 3]. In Sweden, PCC stands as a pivotal element in the “Good quality, local healthcare reform” [4]. This reform necessitates substantial organisational changes to ensure integrated, proactive, and health-promoting PCC across various care settings, responsive to each person’s resources and needs. PCC has been developed to frame care in a holistic and ethical way by establishing a partnership between health and social care professionals and persons in need of care. The concept of PCC is based on ethical principles and has its roots in the holistic paradigm, which highlights the importance of knowing each person as a capable human being with needs and resources [5–7].

The Gothenburg Centre for Person-Centred Care (GPCC) has developed a PCC framework for applying PCC, i.e., PCC practices, serving as a lens for embodying ethical values, guiding professional actions, and enhancing well-being and work performance [6]. This framework describes a model, summarising PCC into three main practices [6, 8]:

1. Initiating personal narratives to get to know each patient as a person, to identify their previous experiences, present situation, needs, abilities, and resources.
2. Co-creating a personal plan in line with identified resources and barriers combined with medical, health, and social research evidence.
3. Documenting and monitoring the plan and adapting it to changes in the goals and/or other circumstances.

Previous research evaluating PCC has to a large extent focused on patients with chronic conditions, showing that PCC could, e.g., improve patients’ self-efficacy, symptom control, satisfaction with care and reduce length of hospital stay and healthcare costs [5]. PCC practices have also shown positive associations with work-related health among health and social care professionals, but the vast majority of the studies are performed in residential care and have mainly addressed registered nurses and nurse assistants [9]. What is less known is to what extent PCC practices are applied, and what impact they have on health and social care professionals’ work-related health and job satisfaction across diverse health and social care settings. This project, PCC@Work, is developed to fill this knowledge gap, focusing on the impact and experiences of applying PCC practices in hospital wards, primary care units, and municipal care.

The work environment for health and social care professionals is characterised by demanding conditions, including high workloads, low control, ethical dilemmas, unclear roles, and demanding schedules which may lead to increased stress and job dissatisfaction [10–13]. Additionally, an unsatisfactory and stressful work setting, along with ethically challenging situations, often prompt health and social care professionals to seek alternative employment [10, 12, 14, 15]. Notably, it is concerning that both newly graduated and experienced professionals show a significant likelihood of considering leaving their current positions [10, 12, 16]. The shortage of skilled professionals has detrimental effects on the workload, quality of care and patient safety [10, 13, 17, 18]. This situation is untenable and requires immediate attention to ensure adequate staffing in the future of health and social care.

In response, PCC@Work aligns itself with the overarching goal of promoting health and well-being in the workplace, aiming to proactively address mental health challenges and mitigate sickness-related absences. One potential remedy is transitioning towards more PCC practices, which could reduce ethical stress and foster more meaningful human interactions in health and social care [7, 19]. PCC practices reportedly foster a heightened ethical consciousness regarding the quality of care, grant greater control over daily tasks, and encourage social collaboration [20]. This is supposed to empower health and social care professionals to align their actions with their personal and professional values by effectively organising and coordinating care with both colleagues and patients [21]. However, adopting PCC practices may also present challenges, particularly due to time constraints, with barriers including traditional culture and practices, sceptical attitudes, structural factors, the time-consuming nature of actively listening to patient narratives, and engaging in the co-creation of health and social care plans [22].

A recent review from our research group [23] illustrates how the introduction of a new professional role through PCC practices could lead to feelings of disorientation and uncertainty among health and social care professionals. These feelings might initially increase stress, and repeated measures with a longitudinal design are therefore essential to show if PCC practices could influence work-related health and job satisfaction in the long run. Significantly, the results showed positive experiences of job satisfaction, including a sense of meaningfulness, enhanced relationships between professionals and persons in need of health and social care, as well as increased appreciation and collaboration [23]. These findings, in combination with the findings from a previous review [9], prompt an inquiry into the degree to which the outcomes were influenced by the specific context of applying PCC practices. This underscores the imperative for comprehensive research in diverse health and social care settings, employing both quantitative and qualitative methodologies, to assess the impact and experiences of PCC practices from the professionals’ perspective. We hypothesise that a development towards increased PCC practises may enhance the work-related health of health and social care professionals, potentially mitigating sources of stress, excessive workloads, and job dissatisfaction.

Providing a comprehensive and transparent protocol is crucial as it enables the conduct and evaluation of research projects by effectively communicating pertinent information to key stakeholders. As such, our intention with this protocol is to convey the complexity of the design of this multi-method project. The longitudinal aspect of the PCC@Work project will ensure that the complex relationship between PCC practices, work-related health, and job satisfaction is thoroughly researched so that fluctuations over time can be captured. This allows for the impact of PCC practices to be monitored and evaluated. Development of PCC may increase health and social care professionals’ perceived levels of stress at an early stage.

## Methods/design

### Project aim

The overall aim of PCC@Work is to follow, describe, assess, and explore the impact of PCC practices in hospital wards, primary care centres and municipal care on health and social care professionals’ work-related health and job satisfaction.

### Study design and setting

**Fig. 1:**
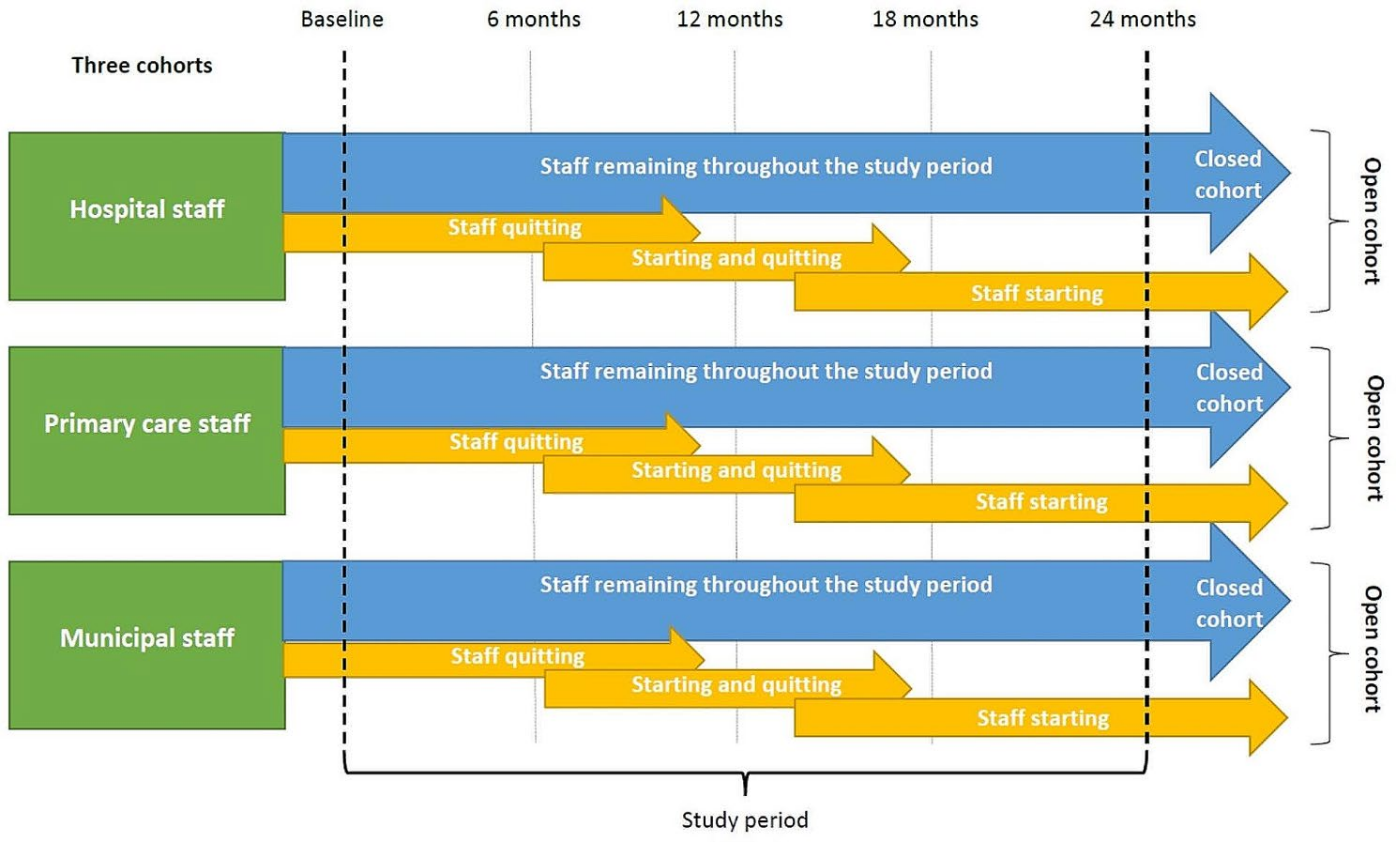
Open cohort study design. The blue arrows represent participants remaining at each care setting during the study period, and the yellow arrows represent examples of several possible scenarios for participants starting and quitting at each care setting during the period

This project has a multi-method design combining a prospective, longitudinal dynamic cohort study with qualitative studies. A web-based questionnaire will be distributed on five occasions within two years to health and social care professionals in hospital wards, primary care centres, and municipal care in Sweden. Employing dynamic cohorts allows participants to leave and enter during the study period. Theoretically and pragmatically, dynamic cohorts are a relevant choice in this project in which we monitor the gradual development of PCC practices. With dynamic cohorts, we can follow the participants at several data collection points. The design allows us to perform repeated cross-sectional analyses using the entire, dynamic, cohort (hospital wards, primary care centres and municipal care) at each data collection point. These different data collection points can be used as cross-sectional studies but can also be compared and give data on changes over time. Moreover, it is possible to make longitudinal analyses by creating a fixed closed cohort identified within the open cohort, to follow participants that stay at the same workplace during the study period. The study design is illustrated in Fig. 1.

The multi-method design includes focus group discussions [24] to generate qualitative data from health and social care professionals from the three settings. Based on a social constructivist approach, the focus groups aim to capture the collective understanding of work-related health and job satisfaction in relation to PCC practices among health and social care professionals in diverse care settings. In addition, there is room for individual interviews with key participants to create a deeper understanding of social processes and contextual influences related to PCC practices through grounded theory [25].

### Participants and recruitment process

PCC@Work addresses health and social care professionals working directly with persons in need of health or social care in hospital wards, primary care centres, and municipal care. Study participants are reached through their work e-mail addresses provided to the research group by each care organisation. All potential participants receive an e-mail, including detailed information on study design, what participation entails, and ethical topics such as voluntariness, consent, and the possibility to withdraw at any time without any negative consequences to participants’ employment. The e-mail also includes a personal link to a web-based questionnaire. Three reminders are sent to participants to facilitate and promote participation. The web-based questionnaires are operated in collaboration with a company with vast experience in using web-based questionnaires.

There is a baseline data collection for the longitudinal study which will have followed-up after six, 12, 18 and 24 months. The impact of PCC practices will be measured after the third (12 months follow-up) and fifth data collection point (24 months follow-up). Repeated cross-sectional analyses will be conducted at each data collection point to explore associations between PCC practices, work-related health, and job satisfaction. In addition, a subsample of these participants will be asked to participate in the focus groups.

### Prospective longitudinal cohort study measures

#### Exposure variable

The *Person*-*Centred Care Assessment Tool* (P‐CAT) [26] is chosen as the exposure variable representing self-reported levels of PCC. P-CAT comprises 13 items aimed at capturing the extent to which professionals perceive PCC practices in their daily work [26]. P-CAT consists of two subscales: the extent of personalising care (EPC; 8 items) and organisational and environmental support (OES; 5 items) [26, 27]. A 5‐point scale from 1 (completely disagree) to 5 (strongly agree) is used for evaluation purposes. The sum score ranges from 13 to 65, with a high score indicating a greater extent of perceived PCC. P‐CAT has shown satisfactory validity and reliability in a Swedish aged care context [27], and has recently been modified by our research group for use in other care settings showing good internal consistency [28].

### Outcome variables

The *Stress of Conscience Questionnaire* (SCQ) [29] is a 9-item measurement for assessing stressful situations and the degree to which they trouble the conscience. This questionnaire was designed in Sweden to explore perceived stress related to not providing the care or activities one wants to provide within a care setting [29]. It consists of nine items, each divided into two parts: an A question that evaluates the frequency of a selected stressful situation using a scale ranging from 0 (never) to 5 (every day), and a B question that evaluates the perceived degree of troubled conscience generated by the situation using a scale ranging from 0 (no troubled conscience at all) to 5 (a very troubled conscience). The A score is multiplied by the B score to reflect the total stress of conscience, ranging from 0 to 25 for each item. Adding the scores for all items gives a total score ranging from 0 to 225. A higher total score signifies a higher perceived stress of conscience. Satisfactory psychometric properties have been reported for the SCQ in a Swedish healthcare population [29].

*The Copenhagen Psychosocial Questionnaire* (COPSOQ III) [30] is a widely used and scientifically tested questionnaire for examining the organisational and social work environment, covering a broad range of domains which can be adopted depending on the aim [30]. We focus on the COPSOQ III domains Demands at Work (6 items), Work Organisation and Job Contents (6 items), Interpersonal Relations and Leadership (7 items), and Work-Individual Interface (5 items). These domains include questions on job strain, demand/control, job satisfaction, meaningfulness, and intent to leave. The item response alternatives correspond to a five-point Likert scale where the mean score between 0 and 100 is calculated for included scales. If > 50% of item responses in a scale are not recorded, the scale measurement will be considered missing. Studies across different professions have corroborated the internal consistency reliability and construct validity of the scales [30].

*The Work Ability Index* (WAI), developed by [31], has been widely used in research in different countries and settings and can be used to assess self-reported individual work ability regarding perceived resources, health, and physical and mental demands [32]. Four out of the seven items from the WAI are applicable to this project and included in the questionnaire. The index has shown very good predictive abilities for measuring nurses’ workability [33], and satisfactory values in a general Swedish population [34].

*The Capacity to Work index* (C2WI-cmd) [35] was developed for assessing capacity to work in relation to common mental disorders in general working populations. C2WI-cmd consists of 12 items. The items include statements covering the capacity to work the last week. The respondent reports to which degree they agree, with the five response alternatives ‘Not at all’; ‘To a low degree’; ‘To a moderate degree’; ‘To a high degree’; or ‘Not relevant’. Our research group tested the C2WI-cmd for reliability, validity and user-friendliness in a Swedish working sample including healthcare professionals [35].

*The WHO mental well-being index* (WHO-5) [36] is a measure of how the respondent has felt in the last two weeks regarding the more positive aspects of their emotional state. Increasingly, well-being has been shown to be important in relation to health and everyday functioning. WHO-5 has shown validity in assessing well-being over time and comparing well-being between groups. Apart from the positive aspects, WHO-5 also prove validity in screening for depression [36].

#### Demographics

Other parts of the questionnaire concern the demographic and confounding factors; gender, age, profession, workplace, type of employment, working hours, shift work, overtime, years working in health or social care and at their organisation, experience and opinion of PCC practices, sickness absence, general health and ongoing implementations or reorganisations in the care setting. All of these will be incorporated into the analyses.

### Statistical analyses

Statistical analysis will focus on repeated cross-sectional and longitudinal analyses to assess changes over time within groups. Statistical analysis will be done at each data collection point. Regression analyses (linear, ANOVA, ANCOVA) will be applied. The primary efficacy analysis centres on the baseline to two-year change in SCQ with the P-CAT as exposure, with Fisher’s non-parametric permutation test for paired observations. Results will be presented at a 5% significance level on aggregated levels.

### Power calculation

For the quantitative studies, a change of 5 units in the *Stress of Conscience Questionnaire* (SCQ) was considered an acceptable effect, in each of the three cohorts (hospital wards, primary care centres, and municipal care), from baseline to 24 months with a power of 80% with Fisher’s non-parametric permutation test for paired observations, and a significance level of 0.05. Thus, 285 health and social care professionals must be included in each of the three cohorts (= 855 in total). We expect a response rate of approximately 40% and therefore aim to invite a minimum of 2200 health and social care professionals at baseline to allow for both staff turnover and withdrawals. The standard deviation for change in SCQ (total score range from 0 to 225) has been estimated to be 30 based on the literature [37].

### Qualitative studies

To allow for a deeper and broader understanding of PCC practices in relation to work-related health and job satisfaction, the e-mail sent out for the 12-month follow-up data collection will invite participants to focus group discussions. Participants interested in contributing to a focus group discussion will then be contacted by the research group for more detailed information and for setting up a time and place for the focus group. Homogeneity will be strived for in terms of care setting and profession, and heterogeneity will be strived for in terms of work experience, national background, age, and sex, to capture a diversity of experiences and broaden the discussions [24].

For the focus group discussions, our intention is to conduct at least two focus groups with health and social care professionals per care setting (a total of at least six focus groups), and we will strive for four to six professionals per group (*n* = 24–36). For the grounded theory study, an open sampling of approximately 15–20 health and social care professionals is estimated.

The focus groups will preferably be conducted in a venue accessible for the participants, or digitally if needed, and they are expected to last 60–90 min. Led by a moderator and co-moderator, discussions centre on key questions formulated by the research group to align with the study’s aim. The moderator guides the discussion, while the co-moderator observes, takes notes, and asks follow-up questions. Sessions begin with an introduction to the study’s aim and structure, followed by open-topic discussions. The moderators’ role is to ensure participant engagement, identify common themes, and pose specific questions to deepen the discussions. All sessions will be audio-recorded and transcribed for subsequent analyses. The grounded theory study will have a similar approach.

The focus groups will be iteratively analysed using a method developed explicitly for focus groups [24]. Focus group data will undergo multiple stages of analysis. Initially, repeated listening establishes an overall understanding. Each transcript is then independently examined to capture essential data. Preliminary themes are created by the researchers, guided by the study’s aim. Raw data is categorised, and descriptive statements are formed. Systematising data under themes involves aligning discussions with relevant categories. This continuous process ensures meaningful communication of discussion meanings. Finally, data is summarised and interpreted collaboratively to foster shared understanding. This analytical continuum transforms raw discussions into condensed, interpretable summaries, forming the basis for a collectively agreed-upon final interpretation. In addition, the individual interviews will be analysed by applying grounded theory [25], in which data collection and analysis will be conducted as simultaneous processes characterised by constant comparisons of data.

## Discussion

There are some limitations to this project. Various factors, including time constraints, lack of direct connection to researchers, survey fatigue, and insufficient interest or motivation, impact participation rates in research, particularly among care professionals [39–40]. Moreover, language barriers contribute to lower questionnaire participation rates for persons born outside the country of residence, affecting municipal care, where over one-third of care professionals are foreign-born [41, 42].

For longitudinal research, fixed cohorts are ideal, but the dynamic nature of work-related studies, especially those involving PCC practices, necessitates following groups with similar exposure combinations. Additionally, uneven distribution among health and social care professionals, with assistant nurses being the largest group, poses challenges to achieve representative sampling. Sensitivity analyses comparing assistant nurses with other professionals can address this issue, and oversampling certain groups may be considered.

The definition of PCC varies across organisations and professions, emphasising the importance of using the P-CAT in the questionnaire to establish a common understanding. Ultimately, the study aims to uncover new insights into the impact of PCC practices on work-related health and job satisfaction among health and social care professionals in hospital wards, primary care centres, and municipal care.

## Data Availability

The datasets produced and analysed in this project are not publicly accessible to uphold the confidentiality commitments made to participants during the informed consent process. However, de-identified data can be provided upon reasonable request for review purposes.

## Abbreviations

PCC: Person-Centred Care
GPCC: Gothenburg centre for Person-Centred Care
P-CAT: Person- Centred Care Assessment Tool
SCQ: Stress of Conscience Questionnaire
COPSOQ III: Copenhagen Psychosocial Questionnaire
C2Wi-cmd: Capacity to Work index
WHO-5: The WHO mental well-being index (5 statement version)
